# Reliability of Compartmental Body Composition Measures in Weight-Stable Adults Using GE iDXA: Implications for Research and Practice

**DOI:** 10.3390/nu10101484

**Published:** 2018-10-12

**Authors:** Aimee L. Dordevic, Maxine Bonham, Ali Ghasem-Zadeh, Alison Evans, Elizabeth Barber, Kaitlin Day, Alastair Kwok, Helen Truby

**Affiliations:** 1 Department of Nutrition, Dietetics & Food, Monash University, Notting Hill 3168, Australia; maxine.bonham@monash.edu (M.B.); alison.evans@monash.edu (A.E.); elizabeth.barber@monash.edu (E.B.); kaitlin.day@monash.edu (K.D.); alastair.kwok@monash.edu (A.K.); helen.truby@monash.edu (H.T.); 2 Department of Endocrinology, Department of Medicine, Austin Health, the University of Melbourne, West Heidelberg 3081, Australia; alig@unimelb.edu.au

**Keywords:** adiposity, body composition, dual X-ray absorptiometry, visceral adipose tissue

## Abstract

The aim of this study was to explore the reliability and precision of body compartment measures, in particular visceral adipose tissue, in weight stable adults over a range of BMIs using GE-Lunar iDXA. Weight-stable participants aged 18–65 years had a total body composition scan on GE-Lunar iDXA either on three separate occasions over a three month period (*n* = 51), or on a single occasion for duplicate scans with repositioning (*n* = 30). The coefficient of variation (CV%) and least significant change (LSC) of body compartments were calculated. The CV was higher for all measures over three months (range 0.8–5.9%) compared with same-day precision-scans (all < 2%). The CV for visceral adipose tissue (VAT) was considerably higher than all other body compartments (42.2% three months, 16.2% same day scanning). To accurately measure VAT mass using the GE iDXA it is recommended that participants have a BMI ≥ 25 kg/m^2^, or VAT mass > 500 g. Changes observed in VAT mass levels below 500 g should be interpreted with caution due to lack of precision and reliability. All other compartmental measures demonstrated good reliability, with less than 6% variation over three months.

## 1. Introduction

Dual energy X-ray absorptiometry (DXA) is one of the most accessible, simple, quick, relatively non-invasive measures of body composition [1,2]. It is now widely used in human studies where changes in body compartment composition attains importance [3,4,5,6,7], providing a measure of physiological responses to obesity interventions. DXA is superior to simple anthropometrics such as body mass index (BMI) as an outcome measure in weight management studies, in particular to detect changes in fat and lean body mass. The presence of visceral adipose tissue (VAT), as a component of total fat mass, has become more pertinent in recent years with further understanding of the differential roles of subcutaneous adipose tissue (SAT) and VAT, especially in terms of metabolic activity. Therefore, being able to compartmentalize body fat into both SAT and VAT has become more relevant. This has been recently reviewed by Marinangeli and Kassis [8] in relation to weight management studies. To further understand metabolic risks associated with different body compositions it has been recommended to include a fast and valid measure of body compartment changes, such as VAT, in obesity treatment [9].

Deposition of VAT in the android region is strongly associated with an increase in chronic disease risk [10,11]. A metabolically abnormal phenotype has been linked with increased body fat levels in people with healthy weight status [12,13,14], and is suggested to be a result of increased VAT in those individuals [14]. As such, it is important to be able to reliably measure the impact of interventions on VAT levels in a range of body composition types. However, VAT cannot be clearly differentiated from SAT by anthropometric measures alone or other non-invasive measures of body composition [15,16,17]. Until recently, quantifying VAT has relied on more complex and specialist techniques such as CT and MRI scans, both of which have strengths and limitations including higher X-ray dose by CT, higher cost, and they require specialist expertise to interpret findings.

The Lunar iDXA scanner (GE Medical Systems Lunar, Madison, WI, USA), with its CoreScan software that calculates both VAT mass (g) and VAT volume (cm^3^), was the first DXA instrument that reported the ability to quantify VAT. Its testing against the reference method of CT scan was reported by Kaul et al. in 2012 [18]. The calculation of VAT from a non-invasive, low radiation technique therefore provides the opportunity to measure changes in fat components. As such, the GE iDXA is a potentially pivotal tool for monitoring the biological impact and the associated health benefits of lifestyle interventions.

However, to accurately report true biological changes from DXA imaging, a measure of precision (reproducibility) of the scanner is used, commonly reported as a percentage of the coefficient of variation (CV) [7,19,20]. The CV for body composition measurements has been reported to have high variance, ranging between 1% and 17% [21,22], and appears to be dependent on the region of interest being analyzed. A recent study reported on precision of VAT measurements from two consecutive total body scans [21]; the CV range was 5.4–17%, and was BMI-dependent, with increased variability observed in the lower BMI ranges. 

Previous reports on the precision error of DXA scans have not investigated the reliability of measurements longitudinally: the current recommended procedure is consecutive repeat scans on the same day with re-positioning [21,23,24]. Short term precision error studies are likely to find low variation in measurements, as extraneous variables such as changes in machine calibration, instrument-functioning drift, scan analysis, and inter- or intra-operator errors [25] may be minimized or not encountered. However, long term stability is vital for correct interpretation of lifestyle intervention studies that aim to measure true body composition changes induced by the intervention, rather than normal biological fluctuations or instrument variation.

In obesity interventions, assessment and monitoring of body composition changes occur over long periods of time. Therefore, temporal reliability analyses may be more reflective of study procedures, allowing better interpretation of results for body composition over usual intervention periods [26]. Since there is no body composition phantom available for visceral fat variability measurement, the longitudinal stability of the GE iDXA instrument for body composition measurements, in particular VAT mass, is crucial for correct interpretation of findings.

This study aimed to assess the variability of body composition measures of weight-stable adults, taken on the GE iDXA. It did this through the assessment of the CV (%) and least significant change (LSC) of measurements for three repeated total body scans over three months, which is the minimum period typically required to measure clinically significant changes in body composition in response to nutrition and lifestyle interventions. Additionally, the precision of the same instrument using the standard protocol of two repeated scans on the same day with subject re-positioning was also performed. We hypothesized that the variability between measurements over three months for each body segment including total body, android, gynoid and VAT would be higher than those observed for the same-day precision measures.

## 2. Materials and Methods 

### 2.1. Participants

Reliability, three-month study: Sixty-five weight stable adults aged 18 to 65 years were recruited by advertisement posted within Monash University and the local community. Weight stability was self-reported as weight change no more than ±2 kg for at least three months prior to participation. Participants attended the Be Active, Sleep, Eat (BASE) Facility at Monash University, Melbourne, on three separate occasions over a three-month period.

Same-day precision: Thirty adults aged 18 to 65 years were recruited by advertisement posted within Monash University and the local community. Participants attended the Be Active, Sleep, Eat (BASE) Facility at Monash University, Melbourne on a single occasion for duplicate scans.

No incentive was offered for completion of either study but all participants were given a copy of their body composition scan at their final visit. All experimental procedures were conducted in accordance with the Declaration of Helsinki and were formally approved by the Monash University Human Research Ethics Committee (CF13/1432-2013000751; CF15/2790-2015001139) and participants provided full informed consent. 

### 2.2. Sample Size

The sample size was estimated based on previously reported data that investigated the reproducibility of DXA scans [27]. It has also previously been estimated that precision studies recruit at least 30 participants to be scanned twice or at least 15 participants scanned three times [28].

### 2.3. Procedures

Reliability, three-month study: Participants attended the laboratory after an overnight fast between 8 am and 10 am. Each participant was required to attend at the same time of day for each visit. Prior to the DXA scan, participants were required to change in to a gown and remove all radio-opaque objects. Height (Holtain stadiometer to 0.1 cm, Holtain Ltd., Crosswell, Pembrokeshire, UK) and weight (Seca Scale 720 to 0.01 kg, Seca Group, Hamburg, Germany) were measured by standard anthropometric techniques to ensure weight stability throughout the assessment period and for the correct scanning mode to be calculated by the GE LUNAR iDXA Narrow-Angle Dual Energy X-ray Densitometer with SmartFAN™ (GE Medical, Software Lunar DPX enCORE 2012 version 14.0, Madison, WI, USA). Participants were then positioned and scanned according to manufacturer’s guidelines for a total body composition scan. BMI was calculated as weight (kg)/height (m^2^).

Same-day precision: The protocol for the precision study was the same as the reliability study, except that participants were asked to stand-up after the first scan, then lie down again for repositioning for the second scan.

Positioning: Participants were positioned for a total body scan on the scanner using standardized procedures. Briefly, participants were placed in supine position with the longitudinal center of the table bisecting the trunk. All parts of the body were in the scan field, except where the participant did not fit within the scan boundary, where arms were placed outside scan regions to obtain VAT data. The top of the participant’s head was located 3 cm below the top scan line. Participants were asked to slightly extend their chin with hands placed in a neutral position, but with space allowed between arms and trunk. Velcro straps were secured at ankle level to minimize movement for the duration of the scan.

### 2.4. Scan Analysis Procedures

**Figure 1:**
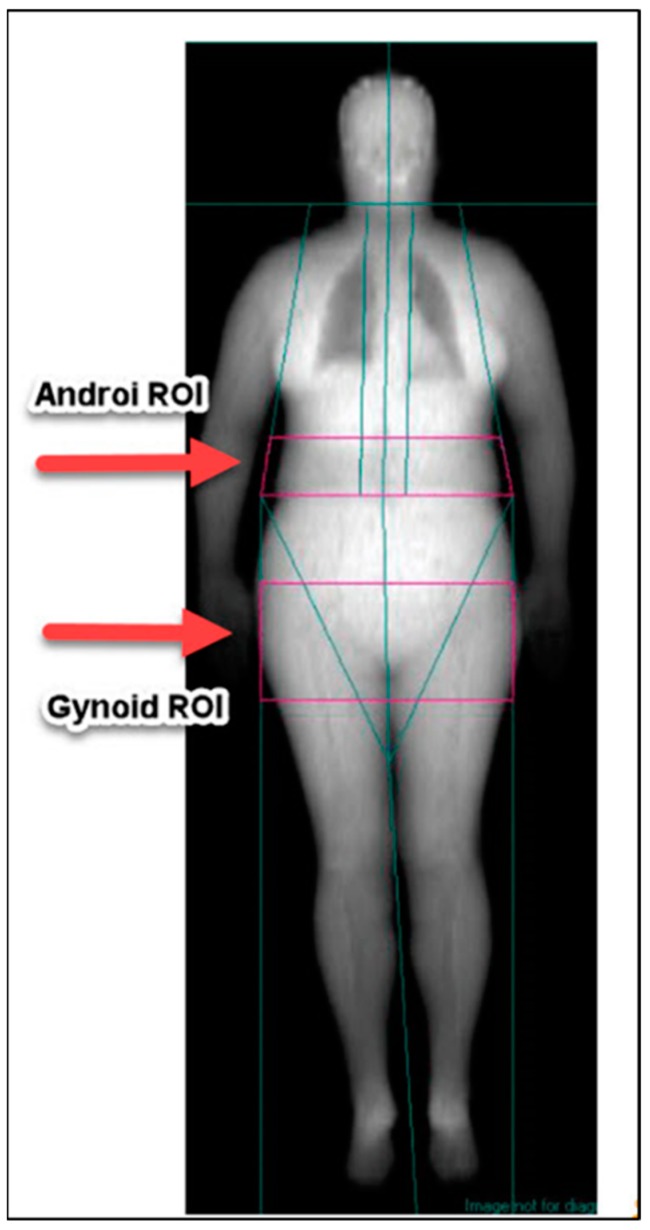
Example scan image with android (top arrow) and gynoid regions (bottom arrow) identified from the GE LUNAR iDXA Narrow-Angle Dual Energy X-ray Densitometer with SmartFAN™ (GE Medical, Software Lunar DPX enCORE 2012 version 14.0, Madison, WI, USA).

All DXA scans and analyses were performed by the same radiographer (AE) who ensured that calibration and phantom scans were performed and passed each day prior to scanning. The manufacturer’s guidelines were followed to ensure the quality of scanning and analyses. Scans were analyzed according to the manufacturer’s instructions. Total body composition was calculated from the whole scan, whereas android and gynoid regions were manually identified by the radiographer (AE) using standard procedures. The android region of interest (ROI) is the area between ribs and iliac crests, the lateral boundaries occur between the trunk and arms, the lower boundary is the top of the iliac crest, and the upper boundary is determined as 20% of the distance between the pelvis and neck cuts. The gynoid ROI is the hip area and includes the area around the proximal femur. The outer leg cuts are the lateral boundaries, the upper boundary is below the pelvis cut by 1.5× android ROI height. The height of the gynoid ROI is determined as 2× android ROI height. Figure 1 shows an example scan including regions of interest. VAT is calculated by the Lunar DPX enCORE software from the android region, determined at L3–L5 level.

### 2.5. Statistical Analysis

Continuous variables are reported as mean and standard deviation (SD) unless reported otherwise. In order to minimize variations that were due to actual changes in body weight and composition, participants who did not meet the criterion for weight stability (±2 kg) for the duration of the reliability study were not included in the analyses. The measurement errors were calculated as CV (%) and the square root of the mean of the sum of the squares of differences (RMSSD) between scans, from which the LSC (at 95% confidence interval) was calculated as recommended by the International Society for Clinical Densitometry [29]. Data were tested for normality using the Shapiro-Wilk test. Differences between groups were assessed by the t-test for parametric data and the Mann-Whitney U test for non-parametric data as reported. For the reliability data, Spearman rank correlation coefficients were calculated for BMI versus VAT mass, and for VAT CV with BMI and fat mass variables. To explore participant characteristics for reliability of VAT measures, a chi-squared test of independence was applied to categories to test whether the variability of measures differed between groups. The categories were determined by applying cut-off levels to the variables in the correlation scatterplots. The cut-off for CV was applied at 10% as the maximum level of acceptable variability, BMI at 25 kg/m^2^, and VAT mass 500 g as the point at which most participants did not exceed 10% variability in repeated measures. To explore the impact of sex, and BMI categories (healthy weight < 25 kg/m^2^, and overweight + obese ≥ 25 kg/m^2^) on the outcome measures, a two-way analysis between groups analysis of variance (ANOVA) was performed. All analyses were performed using IBM SPSS Statistics (Version 22, Armonk, NY, USA).

## 3. Results

### 3.1. Three-Month Reliability Study 

#### 3.1.1. Participants’ Baseline Characteristics

Of the 65 participants, four did not complete the study due to other commitments, and 11 participants did not remain weight stable (±2 kg) over the three-month period and were excluded from the analyses. The height, weight and BMI of the remaining 51 weight stable participants (88% female) are presented in Table 1. The mean age was 35.1 (SD 14.5) years and the median (range) 29.8 (18.7–64.3) years. The CV for body weight was 0.8% over three months.

#### 3.1.2. Body Composition Variability Over Three Months

The android region (from which VAT is calculated) demonstrated the highest variability in all measures of body composition compared with the total body and gynoid region over the three visits (Table 1). The variability of measures in the android region ranged from 2.3% to 5.9%. Whereas the variability between repeated body composition measures that were calculated for the total body, and gynoid region, ranged from 0.8% to 2.8% (Table 1).

Men and women differed in body composition measures: men were taller (*p* = 0.004), weighed more (*p* = 0.013), had decreased gynoid (*p* < 0.001) and total (*p* = 0.009) fat mass, and increased lean mass in all regions (*p* < 0.001). VAT mass was not different between men and women (*p* = 0.321) (Supplementary Table S1). While CV values for women tended to be higher, the levels of variation were not statistically different from men (Supplementary Table S1).

Participants with BMI < 25 kg/m^2^ had significantly lower fat mass and total mass for all compartments (all *p* < 0.01), including VAT mass (*p* = 0.019) when compared with participants with BMI ≥ 25 kg/m^2^. However, analysis by ANOVA did not reveal any significant differences in variability between these groups (Supplementary Table S1).

The CVs for VAT mass and volume were both 42.2%. The measure for VAT mass was detected as 0 g on at least one occasion for seven participants. As such, CVs for VAT mass and volume have also been reported for *n* = 44 (VAT mass > 0 g at all three visits), and remained considerably higher than all other scan measures, both 29.3%. The highest LSC across the three visits for VAT mass and VAT volume was 245.8 g and 260.5 cm^3^, respectively (Table 2). The participants for which VAT was measured as 0 g on at least one occasion had lower BMI (*p* = 0.036), android fat mass (*p* = 0.037), android total mass (*p* = 0.048), as well as VAT mass and volume (both *p* < 0.001) (Supplementary Table S2).

**Figure 2:**
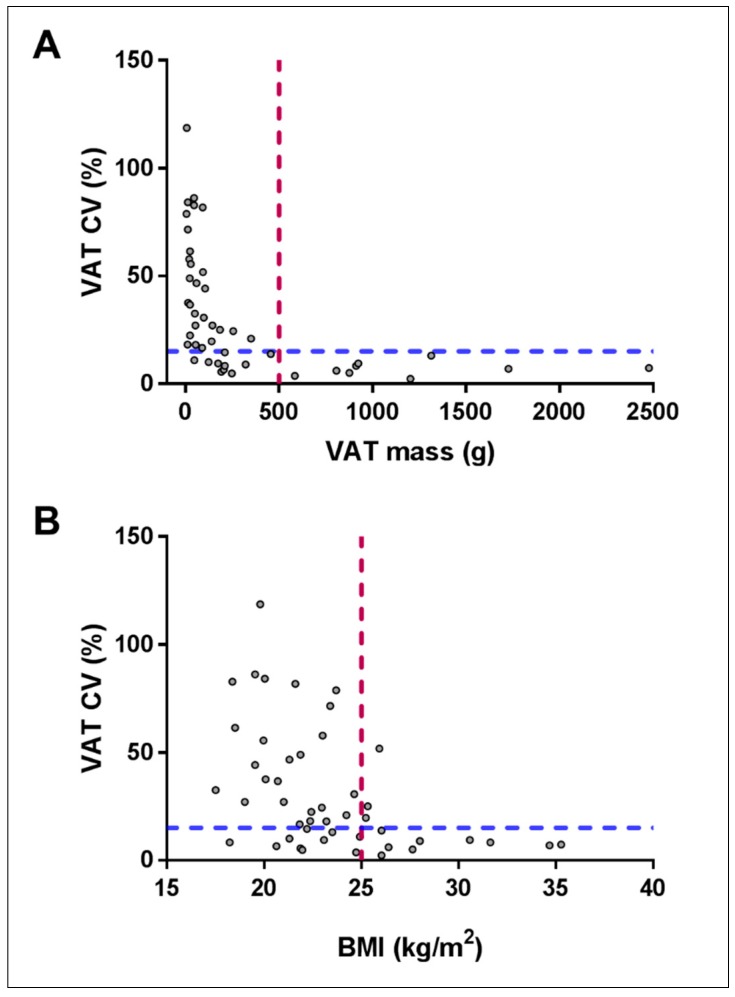
Correlation scatterplot between visceral adipose tissue (VAT) mass coefficient of variation (CV%) and (**A**) VAT mass (g) *r_s_* = −0.795, *p* < 0.001; and (**B**) body mass index (BMI) (kg/m^2^) *r_s_* = −0.537, *p* < 0.001. The dashed lines represent the cut off lines for VAT (500 g) and BMI (25 kg/m^2^) to increase the probability of more reliable CV% for VAT assessments.

BMI was positively correlated with VAT mass (Spearman’s correlation coefficient, *r_s_* = 0.598, *p* < 0.001). The CVs of VAT mass were negatively correlated with weight (*r_s_* = −0.334, *p* = 0.02), BMI (*r_s_* = −0.537, *p* < 0.001) (Figure 2B), total fat mass (*r_s_* = −0.489, *p* < 0.001), android fat mass (*r_s_* = −0.694, *p* < 0.001) and VAT mass (*r_s_* = −0.795, *p* < 0.001, Figure 2A). All correlations indicated that those with higher fat mass demonstrated lower CV for VAT measures over three months. Only 4 out of 12 (33%) participants with a BMI > 25 kg/m^2^ demonstrated CV greater than 10% for VAT, whereas 22 of 35 (63%) participants with a BMI less than 25 kg/m^2^ showed VAT variability greater than 10%. Similarly, participants with VAT mass > 500 g (1 out of 10, 10%) were less likely to have increased variability >10% than people with VAT mass < 500 g (32 out of 38, 84%). People were less likely to have increased variability (>10%) if their BMI was above 25 kg/m^2^ (*p* = 0.001) and VAT mass was over 500 g (*p* < 0.001).

### 3.2. Same-Day Repeated-Scan Precision

Thirty participants, of mean age (SD) 30.9 (10.5) years, median (range) 27.5 (18–49) years, completed the precision study. Height, weight, BMI, and scan data are presented in Table 2. The CVs for all the body compartments for fat mass, lean mass and total mass were less than 2%, whereas the CVs for VAT mass and volume were both 16.2% (Table 2). VAT mass was detected as 0 g on one occasion for one participant, therefore CV is also reported for *n* = 29 for these variables, both 11.8% (Table 2). The characteristics of the participant for which VAT was measured as 0 g were weight 60.7 kg, BMI 22 kg/m^2^, similar to the participants in the three-month study who had measured 0 g VAT.

Men and women differed slightly in body composition measures compared to the three-month cohort; men were taller (*p* = 0.003), had higher levels of android fat (*p* = 0.035), and android (*p* = 0.004), gynoid (*p* = 0.006), and total body (*p* = 0.003) lean mass. VAT mass was higher in men compared with women (*p* = 0.003) (Supplementary Table S1). CV values were not different between men (Supplementary Table S1).

Participants with BMI < 25 kg/m^2^ had significantly lower fat mass, lean mass, and total mass for all compartments (all *p* < 0.01), including VAT mass (*p* < 0.001) when compared with participants with BMI ≥ 25 kg/m^2^. However, analysis by ANOVA did not reveal any significant differences in variability between these groups (Supplementary Table S1).

## 4. Discussion

As little as 5% loss of total body weight can result in clinically meaningful risk reduction in obesity-associated morbidities [30,31]. Body composition, not just weight-status, is an important indicator of disease risk [32] and in particular, increased VAT in the android region [33], even in healthy-weight individuals [12,13,14]. As such, intervention studies may aim to target reductions in VAT mass and consequent metabolic disease risk. Therefore, it is important to be able to reliably measure small compartmental changes over time. The iDXA is generally recognized as a reliable measure of body composition, however as with any laboratory and imaging instrument, outputs can vary over time due to factors such as changes in instrument calibration, fluctuation of component function, and others [34]. Despite stability in total body weight (0.8%), the CVs of android, gynoid and total body fat and lean mass were increased over three months compared with both our own same-day precision data and previously published results [24,35,36]. However, these percentages of variation were still considered within acceptable scientific limits (< 6%) [36,37]. A key strength of this study was that a single radiographer was used to reduce variability associated with different operations and this was confirmed by very low variability in the bone mineral density measures over three months (<2.0% data not shown), and as such the variability in body composition measures reported here were likely due to the long-term variability of the scanner rather than possibly attributed to operator errors or inconsistencies in positioning of participants.

High levels of variability were demonstrated in VAT mass and volume, 11.8% with same day scanning, and greater than 30% over three months. The highest variability over the three month period was observed in participants with a lower volume of VAT. Previously reported same-day precision of VAT on the iDXA, was also increased for participants with a weight in the healthy (BMI 18–25 kg/m^2^) range (17%) compared with participants with overweight and obesity (approximately 5%) [21]. The VAT measures are calculated from the compartmentalization of the android region of the total body scan. All composition measures showed higher variability in this region than the gynoid and total body regions. This is supported by previously published results that demonstrated poor DXA scan reproducibility in the upper body segments compared with the lower regions of the body [36]. As the android region is where the gastrointestinal tract is located, factors that may influence the increased variability include hydration status and whether the participant has consumed food. It is therefore speculated that to increase reliability of scans of this compartment, any variables that may influence its composition be minimized, and as such participants should present to study measurements days fasted and at the same time of day.

A LSC of 260 g for VAT mass was measured, indicating that any changes below this level will not be accurately detectable by iDXA in this cohort. This LSC value must be interpreted with consideration that one of the limitations of the present study of was a higher number of participants within the ‘healthy BMI range’ who demonstrated significantly increased variation in repeated VAT measures compared with participants with BMI ≥ 25kg/m^2^. In future studies that aim to investigate VAT changes in adults with overweight and obesity as defined by a BMI ≥ 25 kg/m^2^ and ≥ 30 kg/m^2^, respectively, the LSC may be lower. Nonetheless, based on the present study, a change >260 g or more is recommended to be confident of a significant change in VAT mass.

Of particular concern was that VAT was detected as 0 g on at least one occasion for seven people in the three-month study, and one person in the precision study. The participants weighed significantly less, and had less fat mass in the android region than participants where VAT was detected on all three occasions. The average BMI was 20 kg/m^2^, and VAT mass, when detectable, was measured to be generally less than 20 g. For the whole sample, measurement of VAT mass was less reliable in participants with a BMI below 25 kg/m^2^. However, as only eleven participants in this study had a BMI over 25 kg/m^2^, it may limit the generalizability of these findings. Regardless, our results indicate that for reliable measurements of changes in VAT using the iDXA, participants need to have a BMI above 25 kg/m^2^. More specifically, the results obtained from the 51 participants who completed the three-month reliability study indicate that reliable measures of VAT mass can only be obtained in people with more than 2 kg android fat mass or 500 g VAT mass. As BMI was positively correlated with VAT mass and negatively associated with variability in VAT, it may serve as a useful indicator when planning nutrition and lifestyle studies aimed at reducing VAT. BMI has previously been shown to predict VAT in young adults; waist circumference may also be a reliable marker [38].

Men and women differ in body composition [39], and the variability of measures over time may be impacted by sex. Adiposity levels in women are associated with menstrual cycle sex hormone patterns [40]; some studies also suggest that body composition is altered during the menstrual cycle [41,42] whereas others have reported no significant impact of cycle phase on body composition measures [43]. The present study did not control for the menstrual cycle of participants; if fluctuations in adiposity were occurring, they may have contributed to the increased level of variability observed between visits. 

The iDXA has previously been validated for the measurement of VAT and demonstrated good agreement between VAT measures with computed tomography (CT) scans in men and women across a range of BMI values [18] and good correlation with MRI [44]. Furthermore, earlier studies have demonstrated that major shifts in VAT can occur in adults with none to modest observed change in body weight [45,46,47]. Therefore, the variability observed in the current study may have been representative of true, normal fluctuation of VAT in weight-stable participants over three months, rather than scan variation. Further studies that compare measures of VAT from the iDXA with scans from CT or magnetic resonance imaging (MRI) to determine accuracy over time are required.

## 5. Conclusions

Despite the CV for total body mass remaining below 1% for both study arms, the variability of measures collected over three months for all body segments was increased compared with repeated same-day scanning. In the case of the variability in VAT, this will make it difficult to assess true loss or gain of VAT during weight loss interventions, particularly in individuals with VAT mass <500 g. 

For future studies that aim to assess changes in VAT mass using the GE iDXA it is recommended that to increase likelihood of accurate interpretation, participants should have a BMI at least 25 kg/m^2^, or VAT greater than 500 g. Data obtained from participants with VAT mass levels below 500 g should be interpreted with caution. Furthermore, we suggest that VAT changes in people with BMI lower than 25 kg/m^2^ would be better to be reported as absolute value rather than percentage changes.

## Figures and Tables

**Table 1:** Participant characteristics and body composition and coefficient of variation by region of interest over three months.

**Number of Participants (Male *n*, (%))**			**51 (6 (12))**							
**Total**	**Visit 1**		**Visit 2**		**Visit 3**					
Height (cm)	165.0 (149.1–178.5)		165.3 (148.8–178.7)		164.9 (148.3–178.3)					
Weight (kg)	59.8 (49.5–83.7)		60.2 (49.2–83.6)		59.7 (47.6–84.0)					
BMI (kg/m^2^)	22.2 (18.6–31.5)		22.3 (18.3–31.7)		22.4 (18.2–31.7)					
**Male**										
Height (cm)	172.0 (167.5–178.5)		171.6 (168.1–178.7)		171.9 (167.8–178.4)					
Weight (kg)	77.6 (69.9–82.5)		75.5 (69.0–81.6)		77.5 (69.4–81.9)					
BMI (kg/m^2^)	25.5 (23.8–28.2)		25.1 (23.3–27.9)		25.5 (23.4–27.9)					
**Female**										
Height (cm)	164.5 (149.1–176.4)		164.3 (148.8–176.4)		164.2 (148.3–176.4)					
Weight (kg)	58.7 (49.5–83.7)		57.9 (49.2–83.6)		58.4 (47.6–84.0)					
BMI (kg/m^2^)	21.8 (18.6–31.5)		21.9 (18.3–31.7)		22.0 (18.2–31.7)					
**Measure (Region of Interest)**	**Visit 1**		**Visit 2**		**Visit 3**		**CV%**	**RMSSD (LSC 95% CI)**		
	**Mean (SD)**	**Range**	**Mean (SD)**	**Range**	**Mean (SD)**	**Range**		**V1–V2**	**V2–V3**	**V1–V3**
A-FM (kg)	1.33 (0.97)	0.27–4.81	1.33 (0.98)	0.30–5.01	1.32 (0.96)	0.25–4.89	5.9	0.10 (0.27)	0.11 (0.31)	0.12 (0.34)
G-FM (kg)	3.53 (1.37)	1.28–7.76	3.54 (1.35)	1.20–7.62	3.55 (1.40)	1.25–8.07	2.8	0.15 (0.43)	0.15 (0.40)	0.19 (0.53)
TB-FM (kg)	18.8 (7.82)	7.62–47.3	18.9 (7.93)	8.10–47.7	18.8 (7.94)	7.81–47.7	2.5	0.73 (2.03)	0.60 (1.67)	1.00 (2.76)
A-LM (kg)	3.01 (0.54)	2.18–4.69	2.98 (0.56)	2.16–4.61	2.99 (0.58)	1.91–4.80	3.1	0.14 (0.39)	0.16 (0.46)	0.14 (0.39)
G-LM (kg)	6.60 (1.29)	4.67–10.2	6.58 (1.30)	4.50–10.2	6.60 (1.31)	4.45–10.1	1.6	0.15 (0.42)	0.17 (0.46)	0.16 (0.43)
TB-LM (kg)	41.0 (7.55)	30.6–61.3	41.1 (7.61)	30.4–61.4	41.1 (7.68)	29.1–62.0	1.2	0.77 (2.14)	0.80 (2.21)	0.86 (2.37)
A-TM (kg)	4.38 (1.23)	2.71–8.51	4.36 (1.22)	2.68–8.42	4.36 (1.27)	2.52–8.45	2.3	0.15 (0.43)	0.14 (0.40)	0.16 (0.43)
G-TM (kg)	10.4 (1.92)	6.81–15.8	10.4 (1.87)	6.86–15.4	10.4 (1.95)	6.65–15.7	1.3	0.22 (0.62)	0.22 (0.60)	0.20 (0.55)
TB-TM (kg)	62.2 (11.9)	43.1–98.2	62.3 (11.9)	43.9–98.9	62.3 (12.1)	42.7–98.7	0.8	0.79 (2.20)	0.67 (1.85)	0.92 (2.56)
A-%Fat	28 (12)	9–58	28 (12)	9–60	28 (11)	10–58	5.5	2.0 (5.5)	2.3 (6.3)	2.7 (7.5)
G-%Fat	34 (9)	16–52	34 (9)	15–51	34 (9)	15–53	2.2	1.2 (3.3)	1.1 (3.1)	1.4 (3.8)
TB-%Fat	30 (8)	17–50	30 (8)	16–51	30 (8)	16–50	2.5	1.1 (3.1)	1.1 (3.1)	1.6 (4.4)
VAT Mass (g)	303.7 (504.9)	0–2540.0	301.4 (516.6)	0–2621.7	280.9 (464.1)	0–2273.1	42.2	50.8 (140.7)	82.7 (229.0)	74.5 (206.3)
VAT Vol (cm^3^)	322.0 (535.2)	0–2692.4	319.5 (547.6)	0–2779.0	297.7 (491.9)	0–2409.5	42.2	66.4 (184.0)	70.2 (194.5)	65.4 (181.2)
*VAT Mass (*g*) ^*	*350.8 (529.1)*	*0.49–2540.0*	*348.3 (542.1)*	*0.46–2621.7*	*324.9 (485.8)*	*2.18–2273.1*	*29.3*	*54.6 (151.3)*	*88.7 (245.8)*	*79.6 (220.5)*
*VAT Vol* (cm^3^) *^*	*371.8 (560.8)*	*0.51–2692.4*	*369.2 (574.6)*	*0.48–2779.0*	*344.4 (514.9)*	*2.32–2409.5*	*29.3*	*57.9 (160.4)*	*94.0 (260.5)*	*84.4 (233.7)*

Participant characteristics are reported median (5th and 95th percentile A = Android). BMI = body mass index; FM = Fat mass; G = Gynoid; LM = Lean mass; CV = coefficient of variation; LSC = Least significant change; RMSSD = Root mean square of the standard deviation; TB = Total body; TM = Total mass; V1 = visit 1, V2 = visit 2, V3 = visit 3; VAT = Visceral adipose tissue; ^ = Included only participants with VAT > 0 g for all three visits (*n* = 44). Italics: Datas are different to above rows.

**Table 2:** Participant characteristics and scan results with coefficient of variation for body composition measurements for participants in the same-day repeated-scan precision study.

**Characteristics**						
Number of participants (Male *n*, (%))		30 (14 (47))		Men		Women
Height (cm) Median (5th–95th percentile)		171.4 (161.9–184.6)		176.6 (169.7–192.3)		165.3 (161.9–184.2)
Weight (kg) Median (5th–95th percentile)		77.8 (51.7–131.9)		102.8 (55.8–145.1)		69.9 (51.7–126.9)
BMI (kg/m^2^) Median (5th–95th percentile)		26.5 (18.9–39.2)		34.2 (18.9–42.53)		24.4 (19.0–37.4)
**Measure**	**Scan 1 Mean (SD)**	**Range**	**Scan 2 Mean (SD)**	**Range**	**CV%**	**RMSSD (LSC 95% CI)**
A-FM (kg)	2.32 (1.77)	0.36–6.56	2.34 (1.81)	0.36–6.60	1.87	0.06 (0.18)
G-FM (kg)	4.75 (2.64)	1.77–11.96	4.79 (2.74)	1.77–12.43	1.28	0.15 (0.43)
TB-FM (kg)	26.80 (15.01)	10.51–60.09	26.86 (15.14)	10.78–60.10	0.74	0.31 (0.85)
A-LM (kg)	3.82 (0.87)	2.32–5.93	3.82 (0.87)	2.29–6.01	1.11	0.08 (0.23)
G-LM (kg)	8.74 (2.21)	5.12–13.46	8.75 (2.23)	5.01–13.59	0.60	0.15 (0.43)
TB-LM (kg)	53.67 (12.54)	32.79–79.23	53.69 (12.46)	32.77–79.93	0.48	0.48 (1.34)
A-TM (kg)	6.19 (2.44)	3.33–11.02	6.21 (2.48)	3.25–11.22	0.63	0.08 (0.21)
G-TM (kg)	13.81 (4.36)	8.60–23.64	13.86 (4.46)	8.57–24.33	0.58	0.18 (0.50)
TB-TM (kg)	83.41 (25.29)	51.58–136.13	83.50 (25.42)	51.60–136.22	0.20	0.40 (1.10)
A-%Fat	33 (15)	8–61	33 (15)	8–61	1.75	0.9 (2.5)
G-%Fat	33 (9)	16–52	33 (10)	16–53	1.02	0.6 (1.7)
TB-%Fat	30 (9)	17–48	30 (9)	17–48	0.80	0.4 (1.1)
VAT Mass (g)	776.9 (808.8)	28.0–2978.2	766.7 (807.5)	0.0–2999.2	16.2	65.5 (181.4)
VAT Vol (cm^3^)	823.5 (857.4)	29.7–3156.9	812.7 (856.0)	0.0–3179.1	16.2	82.2 (227.8)
*VAT Mass (*g*) ^*	*802.4 (810.8)*	*28.0–2978.2*	*793.2 (808.5)*	*23.4–2999.2*	*11.8*	*66.4 (184.0)*
*VAT Vol* (cm^3^) *^*	*850.5 (859.4)*	*29.7–3156.9*	*840.7 (857.0)*	*24.8–3179.1*	*11.8*	*82.5 (228.6)*

A = Android; FM = Fat mass; G = Gynoid; LM = Lean mass; LSC = Least significant change; STM = Soft tissue mass; V1 = Visit 1; V2 = Visit 2; V3 = Visit 3; TB = Total body; TM = Total mass; VAT = Visceral adipose tissue; ^ = Included only participants with VAT > 0 g for all three visits (*n* = 29).

## Supplementary Materials

The following are available online at http://www.mdpi.com/2072-6643/10/10/1484/s1, Table S1 (attachment): Comparisons of body composition and variability for sex and BMI; Table S2 (attachment): Participant characteristics and body composition: Participants with VAT detected as 0 g.
