# Supplementary material for: Reliability of Compartmental Body Composition Measures in Weight-Stable Adults Using GE iDXA: Implications for Research and Practice
Source: Nutrients. 2018 Oct 12;10(10):1484. doi: 10.3390/nu10101484 (PMC6213248; doi:10.3390/nu10101484)
Supplement: Supplementary file 1 [file nutrients-10-01484-s001.pdf]

Supplementary data Table S1

Comparisons of body composition and variability for sex and BMI

**Three month reliability study**					Two-way ANOVA with sex and BMI category (< or >=25) as the fixed variables		
	BMI < 25 kg/m2; n = 39		BMI >= 25 kg/m2; n = 12		**p-values**		
Mean (SD)	Male; n = 3	Female; n = 36	Male; n = 3	Female; n = 9	Main effect: SEX	Main effect: BMI	Interaction: SEX-BMI
Age (years)	38.9 (20.7)	31.1 (12.4)	37.8 (16.8)	48.6 (13.4)	0.804	0.175	0.124
Height (cm)	174.7 (6.0)	164.4 (8.0)	171.6 (0.6)	161.7 (6.6)	0.004	0.394	0.939
Weight (kg)	74.2 (4.7)	56.9 (6.2)	78.6 (3.0)	76.9 (14.9)	0.013	0.002	0.040
BMI (kg/m2)	24.3 (0.7)	21.1 (1.9)	26.7 (1.1)	29.2 (4.0)	0.728	<0.001	0.008
A-FM (kg)	1.5 (0.6)	0.9 (0.4)	1.7 (0.8)	2.8 (1.3)	0.330	0.001	0.004
G-FM (kg)	2.4 (0.1)	3.2 (0.8)	2.9 (1.0)	5.6 (1.5)	<0.001	0.001	0.020
TB-FM (kg)	16.4 (2.6)	15.9 (3.3)	18.0 (6.0)	31.5 (10.4)	0.009	0.001	0.006
A-LM (kg)	3.9 (0.3)	2.8 (0.4)	4.1 (0.6)	3.1 (0.5)	<0.001	0.150	0.654
G-LM (kg)	9.0 (0.5)	6.1 (0.8)	9.3 (1.3)	6.8 (1.0)	<0.001	0.194	0.713
TB-LM (kg)	54.7 (4.6)	38.3 (5.0)	57.0 (7.3)	42.2 (5.8)	<0.001	0.192	0.744
A-TM (kg)	5.5 (0.5)	3.7 (0.4)	5.8 (0.3)	6.0 (1.6)	0.031	<0.001	0.008
G-TM (kg)	11.6 (0.5)	9.5 (1.2)	12.5 (0.6)	12.7 (2.2)	0.130	0.002	0.064
TB-TM (kg)	73.8 (4.7)	56.5 (6.2)	78.0 (3.1)	76.1 (14.6)	0.012	0.002	0.040
A-%Fat	27 (8)	24 (8)	28(12)	45 (1)	0.085	0.007	0.015
G-%Fat	20 (2)	33 (6)	23 (9)	44 (7)	<0.001	0.021	0.140
TB-%Fat	22 (4)	28 (5)	23 (8)	40 (7)	<0.001	0.012	0.026
VAT Mass (g)	665.3 (612.0)	84.3 (90.9)	659.6 (475.1)	904.2 (784.9)	0.321	0.019	0.018
VAT Vol (cm3)	705.2 (648.7)	90.3 (95.6)	699.2 (503.6)	958.5 (832.0)	0.322	0.019	0.018
**Coefficient of variation (CV) %**					Main effect: SEX	Main effect: BMI	Interaction: SEX-BMI
A-FM	5.32 (1.62)	6.77 (3.97)	4.32 (2.08)	3.27 (0.87)	0.900	0.157	0.429
G-FM	3.38 (2.13)	3.04 (2.00)	2.41 (1.51)	2.03 (0.96)	0.663	0.238	0.981

TB-FM	2.91 (1.66)	2.82 (2.52)	1.32 (1.24)	1.37 (0.66)	0.981	0.136	0.945
A-LM	0.90 (0.22)	3.45 (2.22)	2.11 (0.07)	2.78 (1.62)	0.083	0.767	0.307
G-LM	0.80 (0.28)	1.71 (0.91)	0.96 (0.45)	1.37 (0.94)	0.104	0.828	0.526
TB-LM	0.43 (0.34)	1.39 (0.88)	0.64 (0.24)	1.13 (0.58)	0.050	0.947	0.504
A-TM	1.71 (0.59)	2.57 (1.62)	2.04 (0.46)	1.51 (1.14)	0.807	0.588	0.302
G-TM	0.96 (0.57)	1.36 (0.90)	1.06 (0.73)	1.13 (0.52)	0.526	0.874	0.654
TB-TM	0.86 (0.48)	0.91 (0.51)	0.59 (0.35)	0.64 (0.32)	0.810	0.210	0.996
A-%Fat	4.13 (1.24)	6.35 (3.91)	3.65 (2.10)	3.10 (1.25)	0.595	0.234	0.376
G-%Fat	2.46 (2.30)	2.33 (1.76)	2.17 (1.93)	1.72 (1.18)	0.700	0.562	0.834
TB-%Fat	3.33 (1.48)	2.76 (2.34)	1.18 (2.04)	1.32 (0.86)	0.820	0.065	0.709
VAT Mass (g)	15.81 (13.71)	39.70 (30.17)	8.37 (5.74)	15.52 (15.24)	0.202	0.194	0.489
VAT Vol (cm3)	15.82 (13.71)	37.68 (27.47)	8.37 (5.74)	15.52 (15.24)	0.194	0.185	0.507

**Precision study**					Two-way ANOVA with sex and BMI category (< or >=25) as the fixed variables		
	BMI < 25 kg/m2; n = 39		BMI >= 25 kg/m2; n = 12		**p-values**		
Mean (SD)	Male; n = 4	Female; n = 9	Male; n = 10	Female; n = 7	Main effect: SEX	Main effect: BMI	Interaction: SEX-BMI
Age (years)	36.0 (11.6)	34.7 (8.6)	27.3 (9.9)	28.3 (12.4)	0.966	0.072	0.776
Height (cm)	177.0 (5.8)	166.0 (1.9)	177.9 (6.6)	172.3 (10.0)	0.003	0.170	0.309
Weight (kg)	66.9 (12.8)	60.4 (6.9)	110.1 (19.1)	90.3 (26.1)	0.068	<0.001	0.342
BMI (kg/m2)	21.2 (2.7)	21.9 (2.1)	34.6 (4.5)	29.9 (5.2)	0.191	<0.001	0.086
A-FM (kg)	0.9 (0.3)	0.7 (0.2)	4.2 (1.4)	2.6 (1.0)	0.035	<0.001	0.088
G-FM (kg)	1.8 (0.04)	3.1 (0.4)	6.0 (2.0)	6.8 (3.3)	0.185	<0.001	0.760
TB-FM (kg)	12.3 (1.7)	14.2 (1.8)	38.6 (11.9)	34.6 (14.1)	0.784	<0.001	0.448
A-LM (kg)	3.6 (0.8)	3.1 (0.6)	4.6 (0.6)	3.6 (0.6)	0.004	0.006	0.287
G-LM (kg)	8.0 (1.0)	7.0 (1.2)	10.9 (1.5)	8.2 (2.1)	0.006	0.002	0.166
TB-LM (kg)	51.6 (10.4)	43.5 (6.9)	65.7 (8.1)	50.7 (11.3)	0.003	0.005	0.328

A-TM (kg)	4.6 (1.2)	3.9 (0.5)	8.9 (1.7)	6.3 (1.5)	0.004	<0.001	0.076
G-TM (kg)	10.1 (1.1)	10.4 (1.3)	17.3 (3.1)	15.4 (5.1)	0.515	<0.001	0.384
TB-TM (kg)	66.9 (12.7)	60.2 (6.4)	107.6 (16.8)	88.3 (23.8)	0.049	<0.001	0.323
A-%Fat	18 (3)	18 (7)	46 (9)	41 (7)	0.389	<0.001	0.410
G-%Fat	18 (2)	30 (5)	34 (6)	43 (7)	<0.001	<0.001	0.501
TB-%Fat	19 (1)	24 (5)	35 (7)	38 (6)	0.064	<0.001	0.619
VAT Mass (g)	432.0 (354.6)	87.2 (52.1)	1611.4 (773.2)	649.3 (460.0)	0.003	<0.001	0.141
VAT Vol (cm3)	457.9 (375.8)	92.48 (55.2)	1708.1 (819.6)	688.3 (487.6)	0.003	<0.001	0.141
**Coefficient of variation (CV) %**					Main effect: SEX	Main effect: BMI	Interaction: SEX-BMI
A-FM	1.72 (1.48)	2.77 (1.69)	1.37 (0.88)	1.50 (1.00)	0.244	0.116	0.362
G-FM	1.50 (1.63)	1.20 (0.97)	1.17 (1.30)	1.44 (1.22)	0.973	0.919	0.551
TB-FM	1.21 (0.55)	0.79 (0.62)	0.61 (0.44)	0.62 (0.50)	0.324	0.068	0.307
A-LM	0.68 (0.84)	0.64 (0.41)	1.66 (0.82)	1.21 (1.09)	0.439	0.019	0.517
G-LM	0.33 (0.38)	0.89 (0.77)	0.45 (0.46)	0.57 (0.35)	0.126	0.634	0.302
TB-LM	0.47 (0.12)	0.36 (0.20)	0.50 (0.55)	0.61 (0.41)	0.994	0.378	0.487
A-TM	0.53 (0.35)	0.61 (0.55)	0.80 (0.74)	0.47 (0.46)	0.589	0.783	0.383
G-TM	0.49 (0.29)	0.46 (0.55)	0.65 (0.67)	0.70 (0.42)	0.971	0.345	0.855
TB-TM	0.13 (0.03)	0.09 (0.08)	0.25 (0.29)	0.30 (0.21)	0.966	0.049	0.597
A-%Fat	1.80 (1.84)	2.54 (1.13)	1.16 (0.90)	1.57 (1.14)	0.217	0.086	0.723
G-%Fat	1.35 (1.17)	1.24 (0.99)	0.65 (0.71)	1.06 (0.54)	0.639	0.186	0.439
TB-%Fat	1.34 (0.52)	0.86 (0.59)	0.69 (0.47)	0.57 (0.67)	0.186	0.044	0.423
VAT Mass (g)	19.81 (19.03)	18.27 (23.92)	3.52 (3.34)	11.82 (18.71)	0.361	0.109	0.863
VAT Vol (cm3)	19.81 (19.03)	18.27 (23.92)	3.52 (3.34)	11.82 (18.71)	0.361	0.109	0.863

Table S2 Participant characteristics and body composition: Participants with VAT detected as 0 g.

.	**Participants with VAT = 0 g on at Least One Occasion**		**Participants With VAT > 0 g for All Three Measures**		***p*-Value**
Number of participants (Male n, (%))	7 (0 (0))		41 (6 (15))		
Age (years) Median (5th–95th percentile)	24.5 (18.7–46.3)		32.0 (19.2–58.3)		0.336
Height (cm) Median (5th–95th percentile)	165.2 (154.4–178.7)		164.7 (148.7–177.9)		0.310
Weight (kg) Median (5th–95th percentile)	55.2 (47.3–69.3)		60.4 (51.5–83.8)		0.285
BMI (kg/m^2) Median (5th–95th percentile)	19.8 (19.1–23.0)		22.7 (18.4–31.6)		**0.036**
**Measure**	**Mean (SD)**	**Range**	**Mean (SD)**	**Range**	
A-FM (kg)	0.8 (0.2)	0.5–1.2	14.1 (1.0)	0.3–4.9	**0.037**
G-FM (kg)	3.3 (0.6)	2.2–4.1	3.6 (1.5)	1.2–7.8	0.883
TB-FM (kg)	15.7 (3.2)	11.1–20.4	19.3 (8.3)	7.8–47.6	0.299
A-LM (kg)	28.9 (0.5)	2.2–3.5	3.0 (0.6)	2.1–4.7	0.758
G-LM (kg)	62.4 (0.7)	5.1–6.9	6.7 (1.4)	4.6–10.1	0.698
TB-LM (kg)	39.1 (5.3)	30.4–46.4	41.4 (7.9)	30.1–61.4	0.620
A-TM (kg)	3.7 (0.5)	3.2–4.3	4.5 (1.3)	2.6–8.4	0.146
G-TM (kg)	9.8 (1.1)	8.3-11.2	10.5 (2.0)	6.7-15.6	0.528
TB-TM (kg)	57.3 (8.1)	46.9-69.0	63.1 (12.4)	43.2-98.6	0.262
A-%Fat	21 (6)	15–28	29 (12)	9–59	**0.048**
G-%Fat	34 (4)	26–37	34 (9)	15–52	0.920
TB-%Fat	27 (3)	22–31	30 (8)	16–50	0.524
VAT Mass (g)	6.3 (7.1)	0–19.9	341.31 (517.69)	5.0–2478.3	**< 0.001**
VAT Vol (cm^3)	6.7 (7.6)	0–21.1	361.79 (548.75)	5.3–2627.0	**< 0.001**

A = Android; FM = Fat mass; FFM = Fat free mass; G = Gynoid; LM = Lean mass; TB = Total body; TM = Total mass; VAT = Visceral adipose tissue; Differences between groups measured by Mann–Whitney U test for independent samples
